# Heightened fear in response to a safety cue and extinguished fear cue in a rat model of maternal immune activation

**DOI:** 10.3389/fnbeh.2014.00168

**Published:** 2014-05-07

**Authors:** Susan Sangha, Quentin Greba, Paul D. Robinson, Stephanie A. Ballendine, John G. Howland

**Affiliations:** Department of Physiology, University of SaskatchewanSaskatoon, SK, Canada

**Keywords:** fear conditioning, animal model, polyI:C, schizophrenia, reward, prefrontal cortex, hippocampus, amygdala

## Abstract

Maternal immune activation (MIA) during pregnancy is an environmental risk factor for psychiatric illnesses such as schizophrenia and autism in the offspring. Hence, changes in an array of behaviors, including behavioral flexibility, consistent with altered functioning of cortico-limbic circuits have been reported in rodent models of MIA. Surprisingly, previous studies have not examined the effect of MIA on the extinction of fear conditioning which depends on cortico-limbic circuits. Thus, we tested the effects of treating pregnant Long Evans rats with the viral mimetic polyI:C (gestational day 15; 4 mg/kg; i.v.) on fear conditioning and extinction in the male offspring using two different tasks. In the first experiment, we observed no effect of polyI:C treatment on the acquisition or extinction of a classically conditioned fear memory in a non-discriminative auditory cue paradigm. However, polyI:C-treated offspring did increase contextual freezing during the recall of fear extinction in this non-discriminative paradigm. The second experiment utilized a recently developed task to explicitly test the ability of rats to discriminate among cues signifying fear, reward, and safety; a task that requires behavioral flexibility. To our surprise, polyI:C-treated rats acquired the task in a manner similar to saline-treated rats. However, upon subsequent extinction training, they showed significantly faster extinction of the freezing response to the fear cue. In contrast, during the extinction recall test, polyI:C-treated offspring showed enhanced freezing behavior before and after presentation of the fear cue, suggesting an impairment in their ability to regulate fear behavior. These behavioral results are integrated into the literature suggesting impairments in cortico-limbic brain function in the offspring of rats treated with polyI:C during pregnancy.

## Introduction

It has become increasingly apparent that altered neuroimmune mechanisms might play a role in schizophrenia and autism (Brown, 2012; Fineberg and Ellman, 2013; Khandaker et al., 2013). Maternal exposure to viruses during gestation has been associated with increased schizophrenia risk in the offspring (Pearce, 2001; Brown and Derkits, 2010; Brown and Patterson, 2011). These epidemiological studies suggest that MIA alters normal development of the nervous system resulting in cognitive and behavioral abnormalities. Rodent models of MIA during pregnancy recapitulate a variety of behavioral changes relevant to schizophrenia in the offspring including changes in tasks such as prepulse inhibition, latent inhibition, working memory, set shifting, and reversal learning (Meyer et al., 2009; Brown and Derkits, 2010; Brown and Patterson, 2011; Howland et al., 2012; Piontkewitz et al., 2012; Zhang et al., 2012; Dickerson and Bilkey, 2013; Meyer, 2014). Among the core cognitive deficits observed in patients with schizophrenia are disruptions in behavioral flexibility (Pantelis et al., 1999; Brown et al., 2009; Leeson et al., 2009) which are also seen after MIA in the male, but not female, offspring of rats (Zuckerman and Weiner, 2005; Zhang et al., 2012; Savanthrapadian et al., 2013). The behavioral changes observed in rodent models of MIA are likely caused by altered neurodevelopment of cortico-limbic-striatal circuits in the offspring (for reviews see Meyer and Feldon, 2012; Piontkewitz et al., 2012; Dickerson and Bilkey, 2013). As administration of a variety of immune system activators such as the influenza virus, the viral mimetic polyinosinic-polycytidylic acid (polyI:C), and the bacterial endotoxin lipopolysaccharide to pregnant dams produce similar effects in the offspring, factors related to the activation of the maternal immune system are thought to cause the alterations in neurodevelopment and behavior observed in the offspring (Meyer et al., 2009).

Fear conditioning has been studied in the offspring of rodents subjected to MIA during pregnancy. Increased fear responses to context and cue have been reported in the female, but not male, adolescent and adult offspring of mice treated with polyI:C (Schwendener et al., 2009). No differences in fear conditioning between polyI:C-treated and saline-treated rat offspring were found in two studies measuring freezing to auditory conditioned stimuli (Vorhees et al., 2012; Yee et al., 2012) and one study using suppression of drinking in a conditioned emotional response procedure (Zuckerman and Weiner, 2005). Interestingly, dramatic changes in ultrasonic communication were observed in one study during auditory fear conditioning in the absence of observed behavioral differences (Yee et al., 2012). Surprisingly, none of these studies explicitly examined extinction of fear conditioning, which is a form of behavioral flexibility. Patients with schizophrenia show impaired fear inhibition after extinction of cued fear (Holt et al., 2009), an effect that may be due to altered activation of the ventromedial prefrontal cortex (Holt et al., 2012). Thus, the objective of Experiment 1 of the present study was to test the effects of MIA on the extinction of cued fear conditioning. We hypothesized that MIA offspring would not learn to inhibit their fear response after extinction of a learned fear cue.

All studies to date of behavioral flexibility in schizophrenia patients and MIA offspring use tasks designed to test the flexibility of a single behavior. There are no studies assessing flexibility between emotional behaviors, such as fear and reward seeking. Sangha and colleagues recently reported a novel discriminative conditioning task designed to measure the ability to flexibly switch between fear and reward seeking in response to environmental cues signifying fear, reward or safety (Sangha et al., 2013, 2014). This task requires accurate discrimination among multiple environmental cues in order to appropriately respond to potential danger and rewards and thus is more challenging than non-discriminative auditory fear conditioning and involves processing in a neural circuit including the basolateral amygdala (Sangha et al., 2013) and medial prefrontal cortex (Sangha et al., 2014). Therefore, Experiment 2 had two objectives: (1) assess flexibility of fear and reward seeking behavior in a discriminative conditioning task utilizing multiple cues representing fear, reward or safety, and (2) assess inhibition of fear and reward seeking during extinction of learned fear and reward in the offspring of polyI:C- and saline-treated rats. Given the alterations in behavioral flexibility reviewed above and the results of experiment 1, we anticipated that the polyI:C-treated offspring would show impairments in the discriminative conditioning task and its extinction.

## Materials and methods

### Subjects

Timed pregnant Long–Evans dams [gestational day (GD) 7; Charles River Laboratories, Quebec, Canada] were singly housed in transparent plastic cages in a temperature-controlled (21°C) colony room on a 12/12-h light/dark cycle with food (Purina Rat Chow) and water available *ad libitum*. Male offspring of three separate squads of dams were used in the present experiments. All experiments were conducted during the light phase (lights on at 0700 h) and rats were handled for 1 week before commencing experiments. Experimenters were blind to the treatment of the dams and pups during the course of all experiments. All experiments were performed in accordance with the Canadian Council on Animal Care and were approved by the University of Saskatchewan Animal Research Ethics Board. Efforts were made to minimize the number of animals used and their suffering.

### Gestational and neonatal treatment

Treatment methods closely followed those reported previously (Howland et al., 2012; Zhang et al., 2012). On GD 15, dams were individually transported to a room where weight and rectal temperature (Homeothermic Blanket System, Harvard Instruments, MA, USA) were measured. Dams were then anesthetized with isoflurane (5% induction and 2.5% maintenance) and injected intravenously with a single dose of either saline (*n* = 17) or polyI:C (4.0 mg/kg, high molecular weight; InVivoGen, San Diego, CA, USA; *n* = 16) via the tail vein. This procedure took an average of 10 min/animal, and care was taken to ensure the saline-treated dams were anesthetized for the same length of time as the polyI:C-treated dams. Weight and temperature were measured again 8, 24, and 48 h after the injection. Dams were otherwise left undisturbed until the day after parturition. The day of parturition was designated postnatal day (PND) 0. On PND 1, litters were weighed and culled to a maximum of 10 pups per litter (six males and four females where possible). Other than routine husbandry (including taking litter weights on PND 8 and 14), litters were left undisturbed until weaning on PND 21. Weaned male pups from the same litter were housed in same-sex cages of 2–4 animals. Care was taken to ensure that offspring from several litters were included in each group to reduce the influence of litter effects (see figure captions).

### Behavioral apparatus

Standard operant chambers (ENV-008; MedAssociates, St. Albans, VT) encased in sound-attenuating cubicles were used. During Experiment 2, reward pellets were delivered into a recessed port 2.5 cm above the floor in the center of one wall. Port entries and exits were monitored via an infrared beam. Two lights (28 V, 100 mA) located 10 cm from the floor on the same wall as the port served as the 20 s continuous light cue. A light (28 V, 100 mA) located 18 cm above the floor on the wall opposite the port provided constant illumination. Auditory cues were delivered via a high-frequency speaker (ENV-224BM) located 16 cm from the floor on the same wall as the port. Footshock was delivered through a grid floor via a constant current aversive stimulator (ENV-414S). A video camera located on the door of the sound-attenuating shell recorded the rat's behavior.

### Experiment 1: non-discriminative fear conditioning and extinction training

On Day 1 (Figure 2A), rats in Experiment 1 were trained to associate an auditory cue (4 kHz, 20 s, 80 dB) with a footshock (0.8 mA, 0.5 s) that was delivered at the offset of the cue (5 trials, ITI 180 s). On Days 2 (Extinction Acquisition) and 3 (Extinction Recall) rats underwent one session of extinction per day in which the fear cue was presented 20 times without reinforcement (ITI 180 s). The first cue was presented 180 s after the rats were placed in the chamber giving an initial measure of freezing to the context alone.

### Experiment 2: discriminative conditioning and extinction training

Rats in Experiment 2 were trained as described in Sangha et al. (2013, 2014) with some modifications (Figure 3A). Rats were trained to associate an auditory cue (11 kHz, 200 ms on, 200 ms off for 20 s; 70 dB) with delivery of a reward pellet (Dust-less Precision Pellets, 45 mg, Rodent Purified Diet; BioServ, Frenchtown, NJ) (Reward Pre-training; pellet delivered pseudo-randomly between 10 and 20 s after reward cue onset for 25 trials; intertrial interval (ITI), 90–130 s). Food was restricted to 22–24 g of food per day after the third reward learning session. The fifth reward conditioning session included five unreinforced presentations each of the future fear and safety cues in order to habituate the rats to their presentation, thereby reducing any baseline freezing to these novel cues. Rats then had four discriminative conditioning sessions in which the reward cue-pellet association was maintained (pellet delivery at reward cue offset; 15 trials). Another auditory cue (3 kHz, 20 s, 70 dB) was paired with a mild 0.45 mA, 0.5 s footshock and served as the fear cue (shock at cue offset, 4 trials). Footshock intensity was reduced from 0.8 mA (Experiment 1) to 0.45 mA in Experiment 2 because 4 discriminative conditioning sessions consisting of 4 fear trials each were administered resulting in 16 footshock trials over the course of the experiment. In contrast, only 5 footshock trials were presented in Experiment 1. In separate trials the 20 s fear cue was presented at the same time as a 20 s safety cue (one 28 V, 100 mA light located on each side of the port) resulting in no footshock (15 trials). Trials in which the safety cue was presented alone without any footshock were also included (10 trials) to assess whether freezing developed to the safety cue as well as providing the animal with additional trials that contained a safety cue–no shock contingency. Trials were presented pseudo-randomly (ITI, 100–140 s). One day later rats underwent one session of extinction training (Extinction Acquisition) in which both the fear and reward cues were presented pseudo-randomly without reinforcement. The test for Extinction Recall occurred 1 day later.

### Behavioral analyses

In both experiments, fear behavior was assessed offline from videos by measuring freezing, defined as complete immobility with the exception of respiratory movements, which is an innate defensive behavior (Blanchard and Blanchard, 1969). The total number of seconds spent freezing was quantified during the entire 20 s of each cue presentation every 1 s. In Experiment 1, freezing was also scored during the fixed 180 s intervals between each cue presentation and expressed as percent time freezing [(total number seconds during 20 s cue or 180 s interval/20 s or 180 s, respectively) × 100]. In Experiment 2, cue-triggered effects on fear and reward behavior were assessed normalized to the pre-cue period: [(total number seconds freezing during 20 s cue—total number seconds freezing during 20 s immediately before cue presentation)/20 × 100]. A 20 s pre-cue period was chosen due to variable intervals between cues in Experiment 2 (ITI 100–140 s) (Pecina et al., 2006; Morrow et al., 2011; Sangha et al., 2014). Reward behavior during task acquisition was assessed by calculating the percentage of trials in which the rat entered the port, where the reward pellet was delivered upon presentation of the reward cue (Sangha et al., 2013). During reward extinction, reward behavior was assessed by calculating the total time during which the rat's head was in the reward port during the entire 20 s of each cue presentation. This value was then normalized to the pre-cue period: [(total number seconds in port during 20 s cue—total number seconds in port during 20 s immediately before cue presentation)/20 × 100]. Behavioral data were analyzed using one- and two-way repeated-measures ANOVAs and planned comparison paired and unpaired *t*-tests. Behavioral scoring was performed blindly to treatment condition.

## Results

### Effects of polyI:C treatment on dams and offspring

**Figure 1:**
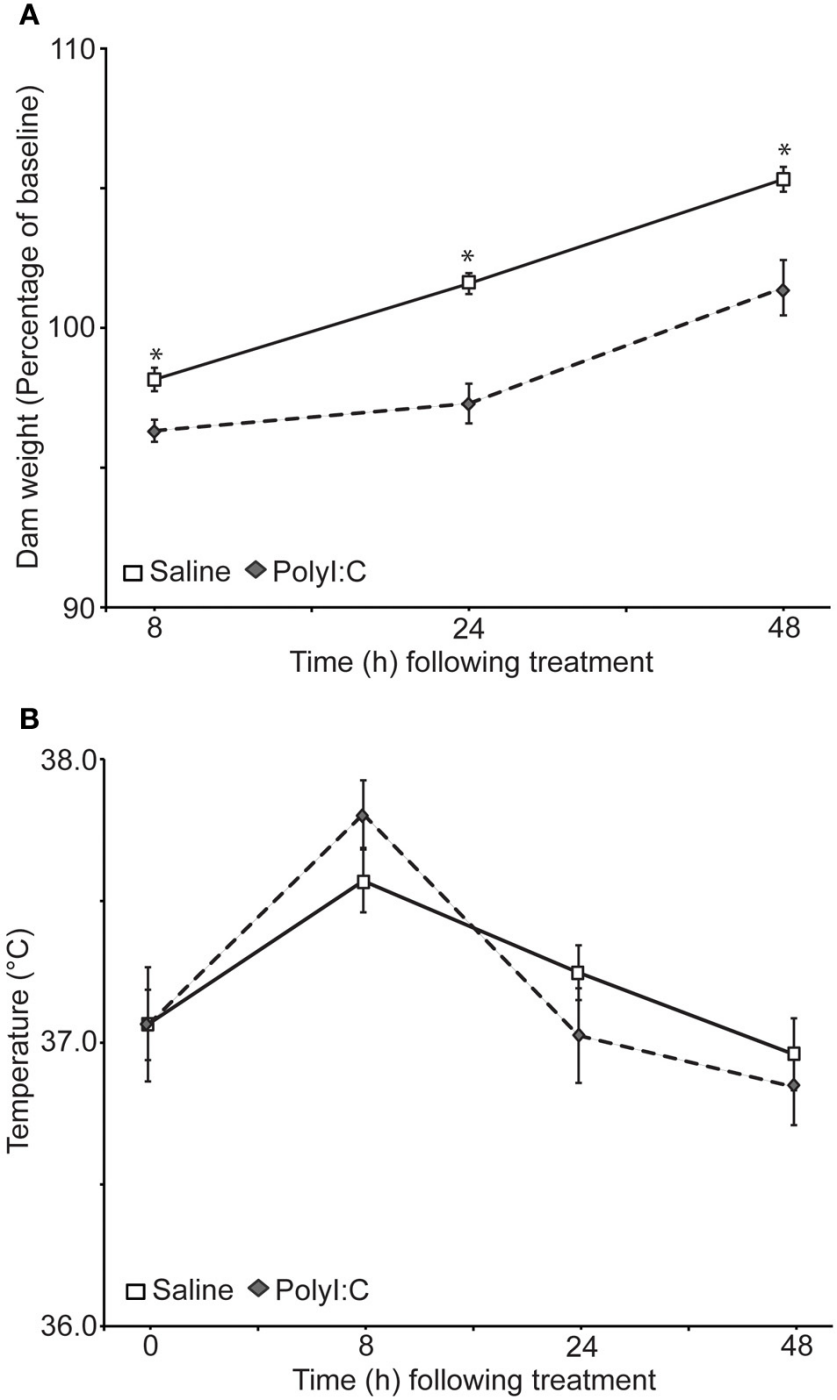
**Effects of gestational day 15 saline- (*n* = 17) or polyI:C-treatment (*n* = 16) on the body weight (A) and temperature (B) of the dams**. Weight and temperature were taken 0, 8, 24, and 48 h after polyI:C injection. **(A)** Weight of the dams as a percentage of their baseline weight. PolyI:C-treated dams displayed significantly less weight gain relative to saline-treated dams, ^*^p < 0.05. The two groups did not differ on initial body weight at time 0. **(B)** Rectal temperature of the dams did not differ significantly between the groups at any time point, although a significant main effect of time was observed. Temperatures were higher in both groups 8 h after treatment.

Weight (Figure 1A) and rectal temperature (Figure 1B) were taken from the dams at 0, 8, 24, and 48 h after treatment with either saline or polyI:C. There was no significant difference in the weight of the dams before treatment [saline: 293 ± 8.85 g; polyI:C 303.38 ± 13.11 g; *t*_(31)_ = −0.66, *p* = 0.51]. Dams from both groups lost weight 8 h after treatment, although dams treated with polyI:C lost significantly more weight. Twenty-four and 48 h after treatment saline-treated dams surpassed their pre-treatment weight, as expected for pregnant rats. PolyI:C-treated dams weighed less than the saline-treated dams at both time points. Results of a repeated measures ANOVA on the percent weight change from baseline showed a significant main effect of treatment [*F*_(1,31)_ = 25.72, *p* < 0.001], time [*F*_(2, 62)_ = 97.73, *p* < 0.001], and a time by treatment interaction [*F*_(2, 62)_ = 4.39, *p* = 0.016]. Rectal temperature changes were observed during the monitoring period with increased temperatures noted in both groups at 8 h after treatment [main effect of time: *F*_(3,93)_ = 28.43, *p* < 0.001]. A main effect of treatment on rectal temperature was not observed in the present sample [*F*_(1, 31)_ = 0.26, *p* = 0.87] nor was a significant interaction between time and treatment [*F*_(3, 93)_ = 2.34, *p* = 0.08].

The number of pups born to either the saline-treated or polyI:C-treated dams did not differ statistically [Table 1; *t*_(31)_ = 0.62, *p* = 0.54]. In addition, a repeated measures ANOVA revealed that the pups gained weight at rates that were not statistically different between the treatment groups from PND1 to PND21 [Table 1; main effect of time: *F*_(3, 93)_ = 990.56, *p* < 0.001; main effect of treatment: *F*_(1, 31)_ = 0.01, *p* = 0.91; time by treatment interaction: *F*_(3, 93)_ = 0.51, *p* = 0.68].

**Table 1:** **Number and weight of the pups born to the saline- and polyI:C-treated dams**.

	**Pups per**	**Average Weight per Pup (g)**			
	**Litter**	**PND1**	**PND8**	**PND14**	**PND21**
Saline	10.82 (0.78)	7.30 (0.12)	20.63 (1.22)	35.54 (1.02)	52.40 (1.07)
PolyI:C	10.13 (0.81)	6.86 (0.23)	19.94 (1.24)	35.03 (1.24)	53.62 (1.26)

*Means are listed with standard error of the mean indicated in brackets. n = 17 litters for saline-treated dams; n = 16 litters for polyI:C-treated dams. PND, postnatal day*.

### Experiment 1: prenatal polyI:C treatment increases contextual freezing during the recall of fear extinction in a non-discriminative cued paradigm

**Figure 2:**
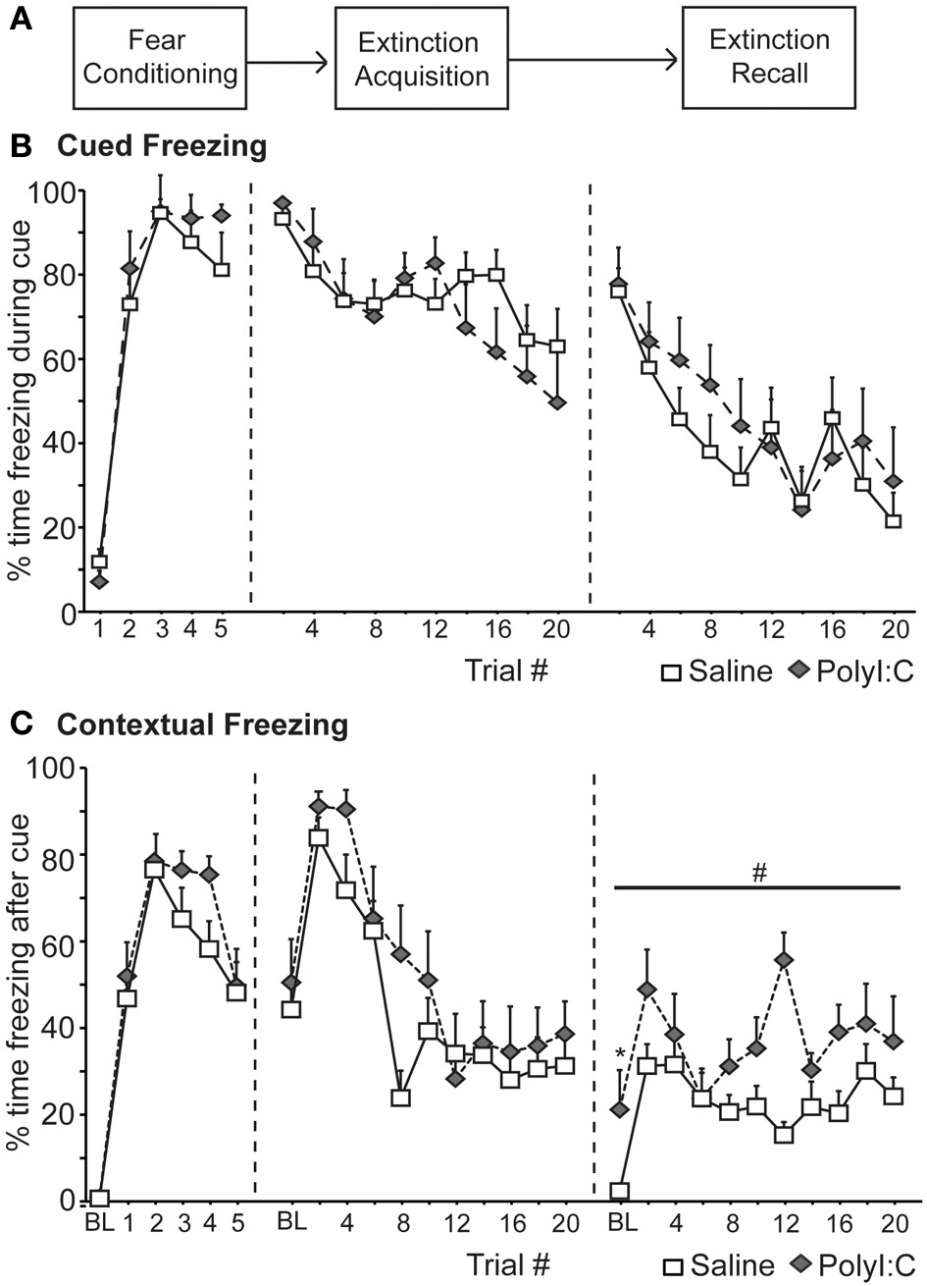
**Deficits in the recall of fear extinction for context in the offspring of rats treated with polyI:C during pregnancy (saline, *n* = 14 from 7 dams; polyI:C, *n* = 10 from 5 dams). (A)** Schematic of experimental design. During Fear Conditioning rats received 5 tone-shock pairings. One day later (Extinction acquisition), the fear cue was presented 20 times without shock in the training context. This was repeated the next day (Extinction recall). **(B)** Cued Freezing. Percent time freezing during blocks of 2 fear cues is shown for each phase of the experiment. No differences between groups were detected. **(C)** Contextual Freezing. Percent time freezing during the 180 s interval after blocks of 2 trials is shown for each phase of the experiment. Also included is the percent time freezing during the 180 s prior to the first cue presentation (baseline, BL). The offspring of polyI:C-treated rats showed higher contextual freezing during Extinction #2 during BL (^*^p < 0.05) and the intervals between the cue presentations (#*p* < 0.05).

We first assessed fear inhibition in MIA offspring after extinction in a non-discriminative cued fear conditioning task utilizing a single cue. Male rats from saline—(*n* = 14) and polyI:C–treated (*n* = 10) dams underwent non-discriminative cued fear conditioning and extinction (Figure 2A). Fear conditioning consisted of five tone–shock pairings; no other cues were presented. Freezing was measured during the cue (Cued Freezing; Figure 2B) as well as during the intervals between cues (Contextual Freezing; Figure 2C). Both saline- and polyI:C-treated rats developed a robust fear response to the cue [*F*_(4, 88)_ = 99.44, *p* < 0.001] with no differences detected between groups [Figure 2B; *F*_(1, 22)_ = 2.18, *p* = 0.15] or a group by trial interaction [*F*_(4, 88)_ = 0.94, *p* = 0.45]. Both groups also displayed similar levels of freezing during the intervals between cues [Figure 2C; group: *F*_(1, 22)_ = 2.21, *p* = 0.15; group by interval interaction: *F*_(5, 110)_ = 0.73, *p* = 0.60] with freezing increasing significantly as the session progressed [*F*_(5, 110)_ = 50.73, *p* < 0.001].

Over the next 2 days the fear cue was extinguished by presenting the fear cue without shock 20 times per day. Again freezing was measured during the cue (Figure 2B) as well as during the intervals between cues (Figure 2C). Significant extinction was observed [Extinction Acquisition: *F*_(9, 198)_ = 7.84, *p* < 0.001; Extinction Recall: *F*_(9, 198)_ = 11.23, *p* < 0.001] and no differences in freezing to the cue were detected between saline and polyI:C-treated offspring during Extinction Acquisition [*F*_(1, 22)_ = 0.51, *p* = 0.48] or Extinction Recall [*F*_(1, 22)_ = 0.91, *p* = 0.35] (Figure 2B). During Extinction Acquisition significant contextual extinction was observed [*F*_(10, 220)_ = 15.29, *p* < 0.001] with no differences observed between groups [Figure 2C; *F*_(1, 22)_ = 2.45, *p* = 0.13]. Differences in contextual freezing were observed between groups during Extinction Recall [*F*_(1, 22)_ = 24.54, *p* < 0.001]. This difference was observed during the 180 s prior to any cue presentation [baseline, BL; *t*_(22)_ = −2.44, *p* = 0.023; Figure 2C] and in the 180 s intervals between all cues [*F*_(1, 22)_ = 20.46, *p* < 0.001; Figure 2C].

In a fear paradigm with a single cue, the male offspring of polyI:C-treated rats display normal fear responses to the fear cue during conditioning and subsequent Extinction Acquisition (Figure 2B). However, polyI:C-treated rats showed increased freezing to the context compared to saline-treated rats during Extinction Recall (Figure 2C).

### Experiment 2a: prenatal polyI:C treatment does not impair discriminative cued fear, safety, and reward conditioning

**Figure 3:**
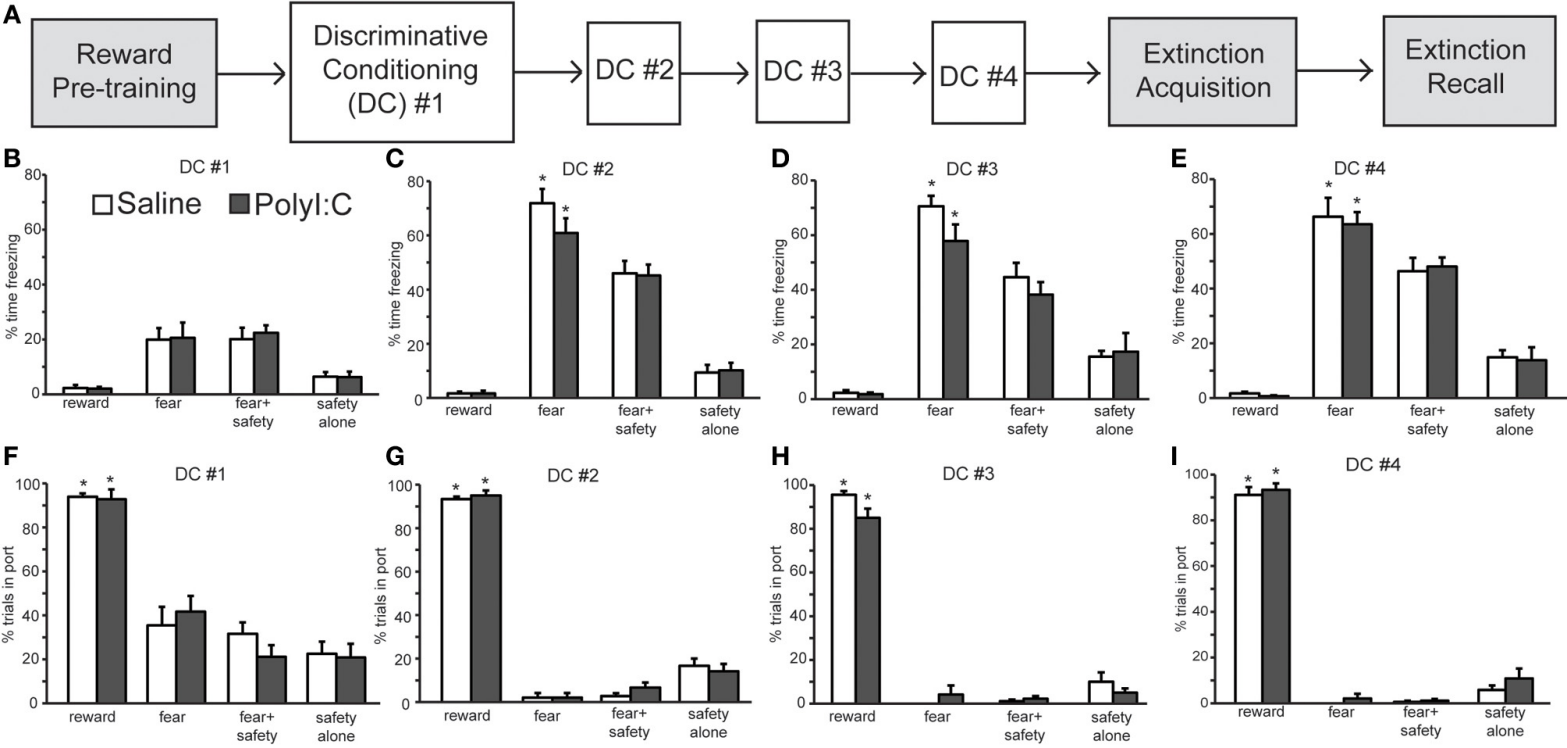
**Normal acquisition and performance of a discriminative conditioning task by both polyI:C- (*n* = 12 from 11 dams) and saline-treated offspring (*n* = 12 from 10 dams). (A)** Schematic of experimental design. Rats were pre-trained on the reward cue-reward pellet association followed by 4 Discriminative Conditioning (DC) sessions in which the reward cue (+reward pellet), fear cue (+footshock), fear+safety cues (no footshock) and safety cue (no footshock) were presented. During Extinction Acquisition all rats received unreinforced presentations of the fear and reward cues. Extinction Recall occurred the next day and consisted of unreinforced presentations of all cues. Data are presented below for the sessions indicated by white boxes. **(B–E)** Percent time spent freezing during each of the cues are presented for each of the DC sessions (#1–4) for each group. Both groups showed freezing levels that were significantly higher to the fear cue than any other cue during DC #2–4. **(F–I)** Percentage of trials the rat entered the port for each of the cues are presented for each DC session. Both groups showed significantly more reward seeking during the reward cue than any other cue during the 4 DC sessions. (^*^p < 0.05, within group analyses).

Experiment 1 assessed regulation of fear behavior in a non-discriminative paradigm. The objective of Experiment 2a was to assess flexibility of fear and reward seeking behavior in a discriminative conditioning task utilizing multiple cues. Male rat offspring from saline- (*n* = 12 offspring) and polyI:C-treated (*n* = 12 offspring) dams underwent discriminative cued fear, safety and reward conditioning (Figure 3A; Sangha et al., 2013, 2014). Both freezing and reward-seeking behavior was measured to the fear cue, fear+safety cues, safety cue alone and reward cue. Each fear and reward cue was reinforced with footshock and food pellet, respectively, at cue offset.

During the first discriminative conditioning (DC) session, little freezing was elicited by any of the cues in the saline- and polyI:C-treated offspring (Figure 3B). There was a significant difference in freezing levels among the cues [*F*_(3, 66)_ = 29.52, *p* < 0.001] but no effect of maternal treatment [*F*_(1, 22)_ = 0.04, *p* = 0.85]. Differential fear responding to the fear cue was observed in the second DC session (Figure 3C). A Two-Way ANOVA revealed a significant main effect of cue [*F*_(3, 66)_ = 187.50, *p* < 0.001] but not treatment [*F*_(1, 22)_ = 0.58, *p* = 0.46]. The percent time spent freezing was significantly greater during the fear cue than during the fear+safety cues, safety cue alone, or reward cue in both saline- and polyI:C-treated offspring (paired *t*-tests, *p* < 0.05). Thus, presentation of the safety cue significantly reduced freezing behavior to the fear cue in both saline- and polyI:C-treated offspring. This reduction of freezing in the presence of the safety cue was maintained in DC sessions 3 [Figure 3D; cue: *F*_(3, 66)_ = 107.54, *p* < 0.001] and 4 [Figure 3E; *F*_(3, 66)_ = 137.01, *p* < 0.001]. No significant differences in freezing levels were detected during any session between the saline- and polyI:C-treated rats for any of the cues [session 3: *F*_(1, 22)_ = 1.17, *p* = 0.29; session 4: *F*_(1, 22)_ = 0.04, *p* = 0.84]. The interaction terms between cue and maternal treatment were not significant for any of the training sessions (statistics not shown). Thus, maternal polyI:C treatment does not impair fear suppression to a learned safety cue in the offspring.

**Figure 4:**
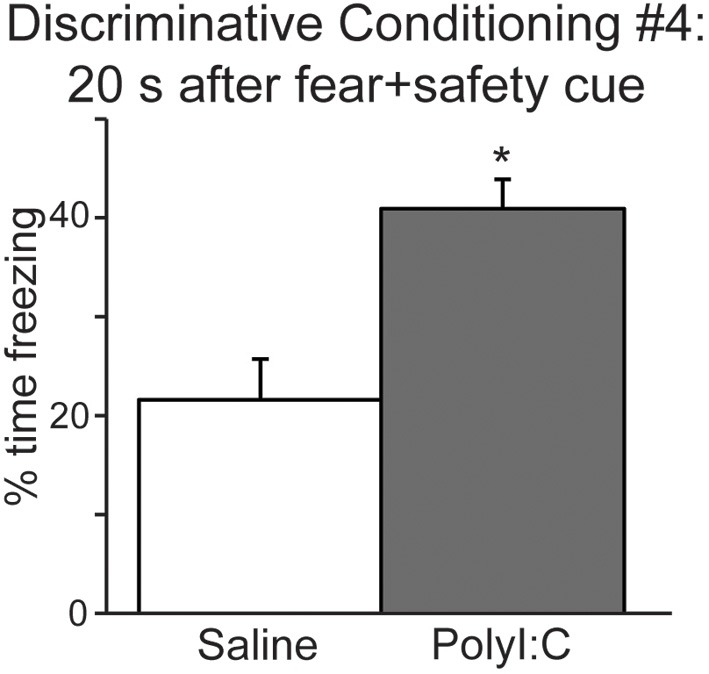
The offspring of polyI:C-treated dams display significantly more freezing during the period following cue offset (20 s) in discriminative conditioning session #4. (^*^p < 0.05).

We also analyzed freezing during the 20 s after the fear+safety cue was presented in DC session 4 (Figure 4) to assess the level of sustained fear after the cue. Percent time freezing during this period remained high for the polyI:C-treated offspring and was significantly greater than saline-treated offspring [*t*_(22)_ = 3.81, *p* < 0.001]. Similar freezing assessments were not made during the 20 s after the fear cue since footshock was delivered at the offset of each fear cue. These data suggest that fear regulation is subtly altered in the offspring following MIA.

Differential reward seeking to the reward cue was observed during all four DC sessions in both saline- and polyI:C-treated offspring. Figures 3F–I show the averaged reward-seeking behavior as measured by the percentage of trials the rat entered the port where the reward pellet was delivered. Two-way ANOVAs revealed significant main effects of cue type during DC sessions 1 [*F*_(3, 66)_ = 89.69, *p* < 0.001], 2 [*F*_(3, 66)_ = 820.11, *p* < 0.001], 3 [*F*_(3, 66)_ = 477.51, *p* < 0.001], and 4 [*F*_(3, 66)_ = 729.06, *p* < 0.001]. The percentage of trials the rat entered the port was significantly greater during the reward cue than during any other cue type across all 4 DC sessions for both saline- and polyI:C-treated rats. Thus, maternal polyI:C treatment does not impair discriminative reward seeking in the offspring. We did not assess reward behavior during the 20 s after the reward cue since each reward cue was reinforced with a food pellet at cue offset; thus, time spent in port after reward delivery most likely reflects consummatory behavior.

### Experiment 2b: prenatal polyI:C treatment increases freezing after extinction of discriminative cued fear conditioning

**Figure 5:**
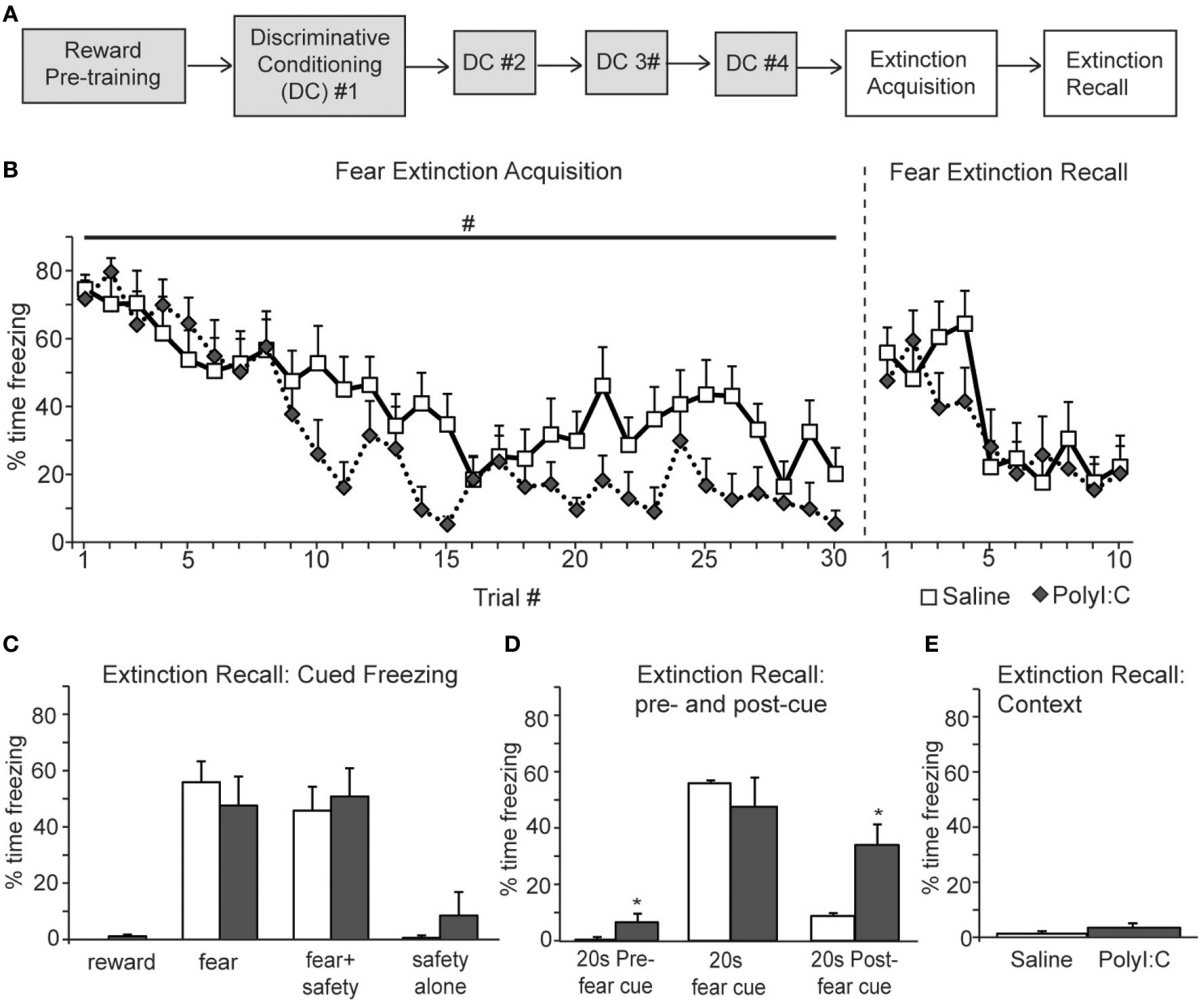
**A, Schematic of experimental design**. Rats were pre-trained on the reward cue-reward pellet association followed by 4 Discriminative Conditioning sessions in which the reward cue (+reward pellet), fear cue (+footshock), fear+safety cues (no footshock) and safety cue (no footshock) were presented. During Extinction Acquisition all rats received unreinforced presentations of the fear and reward cues. Extinction Recall occurred the next day and consisted of unreinforced presentations of all cues. Data are presented below for the sessions indicated by white boxes. **(B, left)** Both groups demonstrated significant within session extinction of freezing behavior to the fear cue. Percent time freezing to each fear cue presentation is shown. Freezing to the last cue presentation was significantly lower compared to the first cue presentation for both groups. There was a main effect between groups with polyI:C prenatally treated rats demonstrating lower freezing levels across Extinction Acquisition (#*p* < 0.05). **(B, right and C)** During Extinction Recall no differences in freezing were detected between groups during any cue. Both groups showed a significant reduction in freezing to the fear cue compared to the beginning of Extinction Acquisition. Panel **(C)** shows freezing during the first presentation of each cue type on the Extinction Recall test. **(D)** polyI:C prenatally treated rats showed significantly higher freezing during the 20 s pre-fear cue and 20 s post-fear cue compared to controls (^*^p < 0.05). **(E)** The percent time spent freezing during the first 180 s of Extinction Recall was not different between groups.

**Figure 6:**
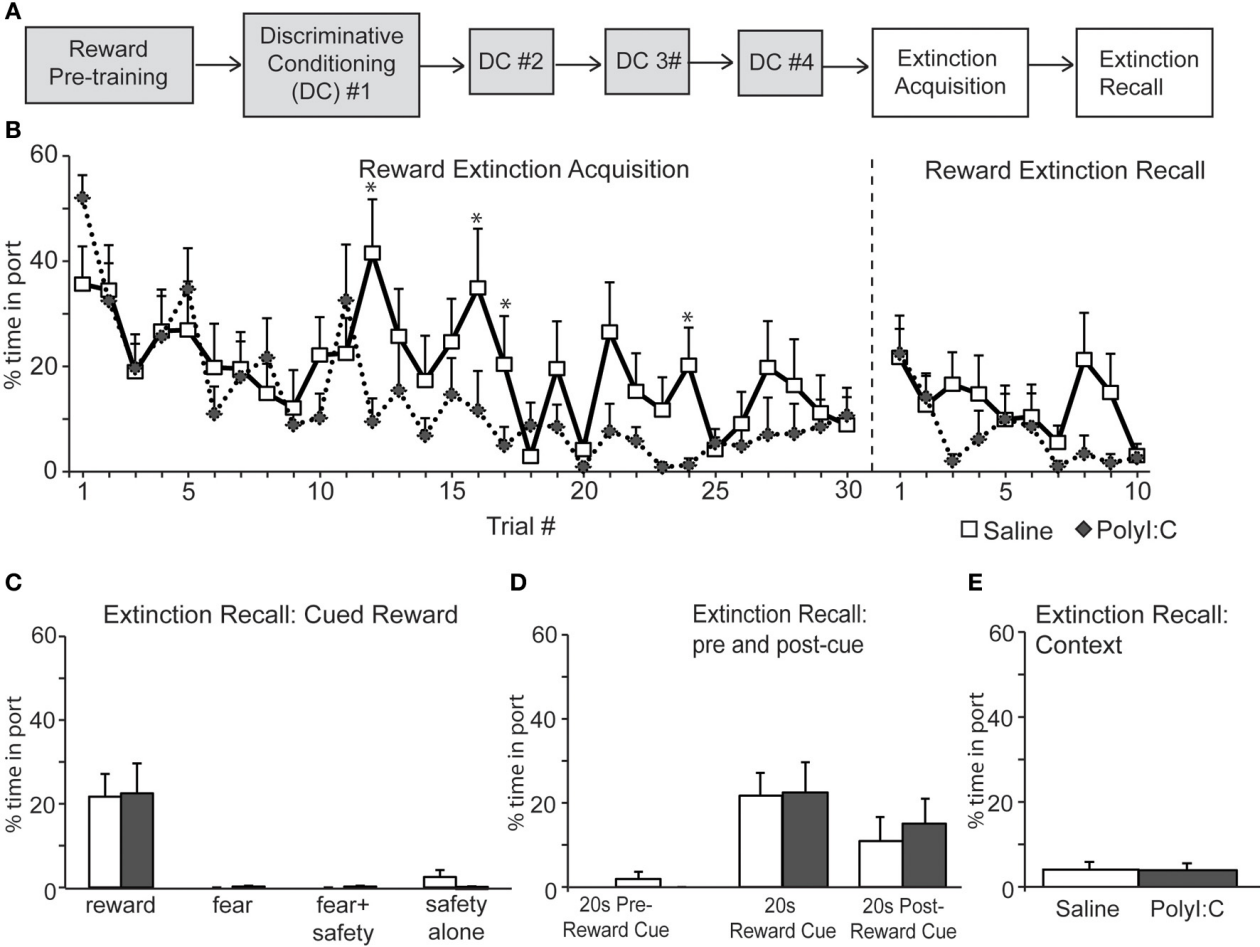
**(A)** Schematic of experimental design. Rats were pre-trained on the reward cue-reward pellet association followed by 4 Discriminative Conditioning sessions in which the reward cue (+reward pellet), fear cue (+footshock), fear+safety cues (no footshock) and safety cue (no footshock) were presented. During Extinction Acquisition all rats received unreinforced presentations of the fear and reward cues. Extinction Recall occurred the next day and consisted of unreinforced presentations of all cues. Data are presented below for the sessions indicated by white boxes. **(B, left)** Both groups demonstrated significant within session extinction of reward-seeking behavior to the reward cue. Percent time in port to each reward cue presentation is shown. Reward seeking to the last cue presentation was significantly lower compared to the first cue presentation for both groups. PolyI:C-treated offspring displayed significantly lower percent time in port during trials 12, 16, 17, and 24 (^*^p < 0.05). **(B, right and C)** During Extinction Recall no differences in reward seeking were detected between groups during any cue. Both groups showed a significant reduction in reward seeking to the reward cue compared to the beginning of Extinction Acquisition. Panel **(C)** shows reward seeking during the first presentation of each cue type on the Extinction Recall test. **(D)** Reward seeking during the 20 s pre-reward cue and 20 s post-reward cue was the same for both groups. **(E)** The total percent spent in port during the first 180 s of Extinction Recall was not different between groups.

Next we assessed inhibition of fear and reward seeking during extinction of learned fear and reward. Subsequent to discriminative cued fear, safety and reward conditioning, both the fear cue and reward cue were extinguished 1 day after the last DC session in a single session in which presentations of the fear and reward cues were intermixed. This “Extinction Acquisition” session was followed by an “Extinction Recall” session 1 day later (Figures 5A, 6A).

Both saline- and polyI:C-treated rats showed significant within session extinction of freezing behavior [Extinction Acquisition, Figure 5B; main effect of Trial, *F*_(29, 638)_ = 11.64, *p* < 0.001]. Percent time freezing was significantly lower during the last fear cue compared to the first fear cue for both groups (planned comparison with paired *t*-tests, *p* < 0.01). There was a main effect between groups, with polyI:C- treated offspring demonstrating lower freezing during Extinction Acquisition [main effect of Treatment, *F*_(1, 22)_ = 7.59, *p* = 0.012]. However, this difference in extinction acquisition curves did not translate into observable differences during Extinction Recall the next day. During Extinction Recall (Figures 5B,C) no differences in freezing were detected between groups for any cue [group: *F*_(1, 22)_ = 0.07, *p* = 0.80]. A significant reduction in freezing to the fear cue compared to the beginning of Extinction Acquisition (paired *t*-tests, *p* < 0.05) was observed for both groups.

When the percent time freezing during the 20 s before and after the fear cue were compared, a significant difference between saline- and polyI:C-treated rats was revealed [Figure 5D; group by cue interaction *F*_(2, 44)_ = 4.25, *p* = 0.021]. PolyI:C treated rats froze significantly more than saline treated rats both before the fear cue as well as afterwards (unpaired *t*-tests, *p* < 0.05). PolyI:C treated offspring show significantly higher freezing levels in the 20 s pre-cue period (6.6 vs. 0.3%). However, it should be noted that the 6.6% pre-cue freezing was mainly driven by 3 animals which displayed freezing levels >10% and that 7 of the 12 polyI:C treated offspring displayed 0% freezing in this pre-cue period. Thus, even though statistically significant, the difference in pre-cue freezing may not be meaningful, particularly in light of the absence of freezing in the first 180 s for both groups. During the postcue freezing period of Extinction Recall, we observed that 10 out of 12 saline rats show <10% freezing whereas only 1 polyI:C-treated rat showed <10% freezing. Thus, most notable is the increased freezing in polyI:C prenatally treated rats during the period after the extinguished fear cue. Even though these rats show significant extinction of freezing to the fear cue, freezing is increased compared to saline-treated rats after fear cue offset. This suggests that despite the significant extinction to the fear cue, the polyI:C-treated rats still display significantly high sustained freezing. This increased freezing in the polyI:C-treated rats was not to the context but dependent on fear cue presentation since the total time freezing during the first 180 s of Extinction Recall was not different between groups (Figure 5E; unpaired *t*-test, *p* > 0.05).

Both saline- and polyI:C-treated rats also showed significant within session extinction of reward-seeking behavior [Extinction Acquisition, Figure 6B; main effect of Trial, *F*_(29, 638)_ = 6.06, *p* < 0.001]. Percent time spent in port was significantly lower during the last reward cue compared to the first reward cue for both groups (paired *t*-tests, *p* < 0.01). No main effect of treatment was found [*F*_(1, 22)_ = 1.78, *p* = 0.20] although a significant Trial by Treatment interaction was observed [*F*_(29, 638)_ = 1.78, *p* = 0.007]. Similar to the Fear Extinction Acquisition data (Figure 5B), polyI:C-treated rats showed significantly higher extinction of reward responding during the reward cue. This effect was restricted to a few trials in the middle of the Reward Extinction Acquisition session (Trials 12, 16, 17, and 24). However, this subtle difference in extinction acquisition did not persist into Extinction Recall the next day. During Extinction Recall (Figures 6B,C) no differences in reward seeking were detected between groups for any cue [group: *F*_(1, 22)_ = 0.02, *p* = 0.90]. A significant reduction in reward seeking to the reward cue compared to the beginning of Extinction Acquisition (paired *t*-tests, *p* < 0.05) was observed for both groups. The percent time spent in port during the 20 s before and after the reward cue was not different between groups [group by cue interaction *F*_(2, 44)_ = 0.24, *p* = 0.79] and the total time spent in the port during first 180 s of extinction recall was also not different between groups (Figure 6E; unpaired *t*-test, *p* > 0.05). Thus, maternal polyI:C treatment does not impair extinction of reward seeking to a reward cue in the offspring.

## Discussion

The present study revealed a number of novel findings regarding the cognitive effects of MIA on the male offspring. In two fear conditioning tasks, we demonstrate that polyI:C-treated offspring express similar acquisition curves as saline-treated controls (Figures 2, 3). Both groups acquired the association between an auditory cue and footshock, as quantified by increased freezing in the presence of the cue. Secondly, both groups demonstrated significant within session extinction to the fear cue in both tasks (Figures 2, 5) and to the reward cue in the discriminative task (Figure 6). PolyI:C-treated offspring showed significantly greater rates of Extinction Acquisition for both fear (Figure 5B) and reward cues (Figure 6B) in the discriminative task. PolyI:C-treated offspring demonstrated impaired fear extinction recall for context, but not cue, relative to saline-treated offspring in the non-discriminative paradigm (Figure 2). During extinction recall in the discriminative paradigm, polyI:C-treated rats displayed low levels of freezing to the context during the baseline period before cue presentation. However, they showed increased freezing in the 20 s before and after the fear cue, but not during its presentation (Figure 5). PolyI:C treated offspring showed a similar high level of sustained fear after the fear+safety cue during discriminative conditioning (Figure 4). Taken together these findings suggest that the offspring of polyI:C-treated dams are impaired in their ability to regulate fear responses in environments learned to be safe.

### PolyI:C treatment significantly reduced body weight of pregnant dams

To assess the acute effects of polyI:C treatment on the dams, weight and rectal temperature were measured (Figure 1). These results and previous findings from Howland's laboratory (Howland et al., 2012; Zhang et al., 2012) suggest that acute polyI:C treatment using this protocol in Long Evans rats consistently reduces maternal weight for at least 48 h afterwards. Others have reported that maternal weight gain is not consistently affected following polyI:C administration in Sprague Dawley rats (Wolff and Bilkey, 2010; Bronson et al., 2011). Weight loss for 1 day has also been described in the Wistar strain although the magnitude of the loss was not quantified (Zuckerman et al., 2003; Zuckerman and Weiner, 2005). Maternal temperature, at least at the time points assessed (8, 24, 48 h after polyI:C administration), does not appear to consistently increase as previously reported in one paper using this protocol (Zhang et al., 2012) but not another (Howland et al., 2012) and the present dataset (Figure 1). Litter size and pup growth was not significantly affected by our treatment protocol (Table 1) as has been previously described for Long Evans, Sprague Dawley, and Wistar rat strains (Zuckerman et al., 2003; Zuckerman and Weiner, 2005; Wolff and Bilkey, 2008, 2010; Howland et al., 2012).

### Nature of the deficit in fear regulation in the polyI:C offspring

The present results using Long Evans rats are consistent with previous reports that MIA during pregnancy does not alter classical fear conditioning in the adult male offspring of either Wistar (Zuckerman and Weiner, 2005) or Sprague Dawley rat strains (Vorhees et al., 2012; Yee et al., 2012). The previous studies assessed freezing to auditory cues and context previously paired with footshock (Vorhees et al., 2012; Yee et al., 2012) and suppression of licking to an auditory cue previously paired with footshock in a conditioned emotional response paradigm (Zuckerman and Weiner, 2005). We report robust freezing to the cue and context on day one during conditioning and day 2 during the initial phase of the first extinction session, which provides a measure of conditioning recall 24 h after training (Figure 2). We used a strong protocol that led to high levels of freezing to both the cue and context during the first extinction session. It should be noted that freezing (approximately 50%) during the baseline period of Extinction Acquisition did not differ between the saline- and polyI:C-treated offspring (Figure 2B). Interestingly, Yee et al. (2012) also showed that fear conditioning was not altered in their model, although a robust alteration in the production of ultrasonic vocalizations occurred during fear conditioning. Specifically, the number of short 22 kHz, but not 50 kHz calls, was increased suggesting that anxiety-related behavior is altered in their model (Yee et al., 2012).

It is noteworthy that in Experiment 2 a lower footshock intensity was used than Experiment 1 (0.45 vs. 0.80 mA) due to the increased number of footshock trials administered in Experiment 2 (5 trials in Experiment 1 vs. 16 trials in Experiment 2). This may have influenced the level of freezing at the beginning of extinction for the two experiment; Experiment 1 showed freezing levels near 100% to the fear cue whereas Experiment 2 showed freezing levels near 75% to the fear cue. Also, Experiment 1 required the animal to regulate its fear behavior to a single cue in the environment whereas Experiment 2 had three cues. Together these factors may explain the differences seen in the acquisition rates of fear extinction between the two experiments.

In the discriminative conditioning task (Figure 3), we observed that both saline- and polyI:C-treated offspring displayed rates of task acquisition similar to those previously reported (Sangha et al., 2013, 2014). During the course of four discrimination sessions, the rats increased the time spent freezing during the fear cue that was paired with shock and showed less freezing when the safety cue was paired with the fear cue. Both groups also maintained reward-related responding only during presentations of the reward cue. Previous studies have shown that polyI:C-treated rats are not impaired on simple discrimination tasks motivated by either food reward (Zhang et al., 2012) or escape from a water maze (Zuckerman and Weiner, 2005). The discriminative paradigm we employ extends these previous findings by showing that MIA does not impair discrimination among cues signifying different outcomes in a single task. In addition, suppression of freezing to a compound fear and safety cue was not modified.

To our knowledge, this is the first study to directly assess extinction of fear conditioning in the offspring of polyI:C-treated rats (Figures 2, 5). In the first experiment, we observed within session extinction of freezing to the auditory cue during both extinction sessions with no differences between the saline- and polyI:C-treated offspring. Freezing to the context during the time periods between cue presentations was also significantly reduced (i.e., extinction was observed) during Extinction Acquisition for both groups. However, during Extinction Recall, the polyI:C-treated offspring showed significantly more freezing during the baseline period before the cues were presented and during the periods of time between the cues. Thus, polyI:C-treated rats have a deficit in the recall of extinction to context.

The task used in the second experiment is more complicated as the rats are conditioned to cues signifying fear, reward, and safety. We observed significantly greater extinction of freezing to the cue in the polyI:C-treated offspring during Extinction Acquisition that did not continue into Extinction Recall 24 h later. Interestingly, polyI:C-treated rats froze significantly more during the periods before and after presentation of the fear cues during Extinction Recall. Most notable was the level of sustained fear evident in the polyI:C-treated offspring after cue offset, suggesting extinction of fear is impaired despite similar freezing levels to controls during the extinguished fear cue. This greater sustained freezing is not only apparent after the fear cue alone in extinction recall but also after the fear+safety cue in discriminative conditioning. Together it suggests that the polyI:C-treated offspring show altered fear regulation in “safe” situations, i.e., in the presence of an explicit safety cue and after extinction of the fear cue.

Freezing levels to both the fear cue and fear+safety cue after extinction were ~50% (Figure 5C) which is similar to the freezing levels seen to the fear+safety cue during the last discriminative conditioning session (Figure 3E). Thus, extinction did reduce freezing to the fear cue to the same level as the fear+safety cue. However, one may expect that the safety cue should further reduce freezing to the fear+safety cue after extinction which we did not observe. Instead the freezing levels to the fear+safety cue after extinction was similar to the amount of freezing to the fear+safety cue during the last discriminative conditioning session (compare Figure 5C to Figure 3E).

### Neural substrates underlying the alteration in fear regulation in the polyI:C-treated offspring

Fear conditioning and extinction depend on a neural circuit including the infralimbic region of medial prefrontal cortex, dorsal hippocampus, and amygdala (Maren and Quirk, 2004; Ji and Maren, 2007; Myers and Davis, 2007; Quirk and Mueller, 2008). Extinction of context memory appears to specifically involve interactions between the medial prefrontal cortex and hippocampus (Corcoran and Maren, 2001). For example, theta coupling between these structures increases during contextual fear conditioning, decreases during extinction, and rises during the initial phase of extinction recall (Lesting et al., 2011). Neural activity in a similar circuit may be involved in safety cue processing (Rogan et al., 2005; Pollak et al., 2008; Christianson et al., 2012; Likhtik et al., 2014). More specifically, Sangha and colleagues showed that a subpopulation of neurons in the basolateral amygdala specifically responded to the fear+safety cues using the same discriminative conditioning paradigm as Experiment 2 (Sangha et al., 2013). In a subsequent paper, the role of the prelimbic and infralimbic sub-regions of medial prefrontal cortex were described using temporary inactivations (Sangha et al., 2014). Consistent with our behavioral findings and the known circuitry underlying fear and reward processing are alterations in the amygdala, hippocampus, and prefrontal cortex of the offspring of rodents exposed to polyI:C during pregnancy (Meyer and Feldon, 2012; Piontkewitz et al., 2012; Dickerson and Bilkey, 2013). For example, the offspring of polyI:C-treated rats show altered synchrony between medial prefrontal cortex and dorsal hippocampus in the theta and low-gamma frequency bands of the electroencephalogram (Dickerson et al., 2010). These changes in synchrony correlate with behavioral changes in prepulse inhibition of the acoustic startle response (Dickerson et al., 2010) and may be directly relevant to the deficits in the recall of the contextual aspects of extinction we observed in the present study.

## Conclusion and implications for neuropsychiatric disorders

The present findings with two tasks that assess conditioned fear, reward, and safety behavior suggest that the male offspring of rats subjected to MIA during pregnancy show impaired fear regulation after extinction. While the treated offspring showed similar task acquisition and extinction learning as control offspring, the polyI:C-treated offspring displayed marked deficits in the recall of extinction to context. Indeed, patients with schizophrenia have normal conditioned fear acquisition and extinction learning but impaired context-dependent extinction recall for a conditioned stimulus (Holt et al., 2009). The findings between the two studies are not entirely consistent as the present data from our experiments showed normal extinction recall for the cued freezing, but not context freezing. Holt and colleagues demonstrated impaired cued extinction recall and impaired context-dependent extinction in a renewal test (Holt et al., 2009). By using functional imaging and the same task design, the deficit in fear extinction recall was shown to correlate with reduced activation of the ventromedial prefrontal cortex (Holt et al., 2012), an area also thought to be involved in safety signaling (Rauch et al., 2006; Sangha et al., 2014). Further understanding of the deficits in conditioned fear and fear extinction in animal models of schizophrenia may prove valuable in understanding similar cognitive changes in psychiatric disorders.

## Author contributions

Susan Sangha designed the experiments, conducted research, analyzed data, and co-wrote the manuscript. Paul D. Robinson conducted research and analyzed data. Quentin Greba designed the experiments, conducted research, and analyzed data. Stephanie A. Ballendine conducted research. John G. Howland designed the experiments, analyzed data, co-wrote the manuscript, and supervised the project.

### Conflict of interest statement

The authors declare that the research was conducted in the absence of any commercial or financial relationships that could be construed as a potential conflict of interest.
